# Chemopreventive and renal protective effects for docosahexaenoic acid (DHA): implications of CRP and lipid peroxides

**DOI:** 10.1186/1747-1028-4-6

**Published:** 2009-04-02

**Authors:** ME El-Mesery, MM Al-Gayyar, HA Salem, MM Darweish, AM El-Mowafy

**Affiliations:** 1 Departments of Biochemistry, Faculty of Pharmacy, Mansoura University, Mansoura 35516, Egypt; 2 Departments of Pharmacology, Faculty of Pharmacy, Mansoura University, Mansoura 35516, Egypt

## Abstract

**Background:**

The fish oil-derived ω-3 fatty acids, like docosahexanoic (DHA), claim a plethora of health benefits. We currently evaluated the antitumor effects of DHA, alone or in combination with cisplatin (CP) in the EAC solid tumor mice model, and monitored concomitant changes in serum levels of C-reactive protein (CRP), lipid peroxidation (measured as malondialdehyde; MDA) and leukocytic count (LC). Further, we verified the capacity of DHA to ameliorate the lethal, CP-induced nephrotoxicity in rats and the molecular mechanisms involved therein.

**Results:**

EAC-bearing mice exhibited markedly elevated LC (2-fold), CRP (11-fold) and MDA levels (2.7-fold). DHA (125, 250 mg/kg) elicited significant, dose-dependent reductions in tumor size (38%, 79%; respectively), as well as in LC, CRP and MDA levels. These effects for CP were appreciably lower than those of DHA (250 mg/kg). Interestingly, DHA (125 mg/kg) markedly enhanced the chemopreventive effects of CP and boosted its ability to reduce serum CRP and MDA levels. Correlation studies revealed a high degree of positive association between tumor growth and each of CRP (r = 0.85) and leukocytosis (r = 0.89), thus attesting to a diagnostic/prognostic role for CRP.

On the other hand, a single CP dose (10 mg/kg) induced nephrotoxicity in rats that was evidenced by proteinuria, deterioration of glomerular filtration rate (GFR, -4-fold), a rise in serum creatinine/urea levels (2–5-fold) after 4 days, and globally-induced animal fatalities after 7 days. Kidney-homogenates from CP-treated rats displayed significantly elevated MDA- and TNF-α-, but reduced GSH-, levels. Rats treated with DHA (250 mg/kg, but not 125 mg/kg) survived the lethal effects of CP, and showed a significant recovery of GFR; while their homogenates had markedly-reduced MDA- and TNF-α-, but -increased GSH-levels. Significant association was detected between creatinine level and those of MDA (r = 0.81), TNF-α ) r = 0.92) and GSH (r = -0.82); implying causal relationships.

**Conclusion:**

DHA elicited prominent chemopreventive effects on its own, and appreciably augmented those of CP as well. The extent of tumor progression in various mouse groups was highly reflected by CRP levels (thus implying a diagnostic/prognostic role for CRP). Further, this study is the first to reveal that DHA can obliterate the lethal CP-induced nephrotoxicity and renal tissue injury. At the molecular level, DHA appears to act by reducing leukocytosis, systemic inflammation, and oxidative stress.

## Review

Cancer ranks second only to cardiovascular disease as a cause of mortality. There are several treatment modalities to control cancer. Unfortunately, surgery and radiation are only effective against local neoplasms. Moreover, chemotherapy tends to indiscriminately destroy both normal and tumor cells, as a consequence of their remarkably low therapeutic windows. The latter profiles trigger an endless series of serious adverse reactions that can be life-threatening. Further, the frequent eruption of resistance to chemotherapeutic agents poses a serious challenge to their efficacy in cancer management. For instance, cispaltin (CP) is a commonly used cytotoxic agent in the treatment of numerous solid tumors [1]. However, its clinical usefulness is limited by the development of a myriad of adverse reactions; including a serious, possibly fatal nephrotoxicity that is reproducible in many animal models [2]. Hence, the search has unfailingly continued to identify more promising antitumor agents or combinations that would preferably exhibit less health hazards [3].

A major aspect of this drug search, therefore, has targeted natural agents; hoping that they would augment actions or reduce doses of ordinary chemotherapeutic drugs; thus improving their overall performance. One of such natural agent candidates has been docosahexanoic acid (DHA), an ω-3 fatty acid that is commonly found in cold-water fish-oil, and some vegetable/algae oils. Naturally, DHA is a major component of brain gray matter and of the retina in most mammalian species and is considered essential for normal neurological and cellular developments [4]. Accordingly, supplemental DHA exhibited a plethora of health benefits; including protection against cardiovascular, neurological and neoplastic diseases. The antineoplastic effects of DHA that have been evaluated in animal models and relevant human trials, showed some hope that this fatty acid may offer anti-tumor profiles, and could also enhance the sensitivity of tumor cells to ordinary chemotherapy [5]. Nevertheless, a paucity of information is available on its possible combined effects with CP. Also, because of its nature as a polyunsaturated fatty acid, the impact of DHA on lipid peroxidation has been controversial [6]. Besides, thus far, the effects of DHA on the *in-vivo *growth of Ehrlich cells (breast-cancer-type cells) have never been sought. Also, the possible diagnostic/prognostic impact of CRP in solid tumor carcinogenesis has been poorly understood. Therefore, in EAC-solid tumor model, we evaluated the chemopreventive effects of DHA, its impact on that of CP, and the utility of c-reactive protein (CRP) as a diagnostic/prognostic predictor of this tumor growth and/or responsiveness to chemotherapy. On the other hand, CP induces substantive nephrotoxicity that is characterized by renal tubular necrosis, proteinuria with upregulation of specific signals like TNF-α, chemokines and cytokines [2]. Therefore, a principal current interest was to elucidate whether, and how then, DHA could alleviate this CP-evoked lethal renal dysfunction in rats.

### Drugs and chemicals

Cisplatin (CP): Vials (10 mg/10 mL, each) were obtained from Merck Co, Egypt. Docosahexanoic acid (DHA): Obtained from Healthspan Co, UK, as capsules. Each provides 100 mg of pure DHA. Rat-TNF-α-ELISA kit was purchased from R&D Systems Inc, Minneapolis, Minnesota, USA).

Trypan blue dye was purchased from Sigma Aldrich Chemical Co, St. Louis, Missouri, USA. A stock solution was prepared by dissolving one gram of trypan blue in 100 mL saline. The working solution was prepared by diluting 1 mL of stock solution with 9 mL of saline. All other chemicals used, acetic acid, calcium chloride anhydrous (CaCl_2_), calcium chloride dihydrate (CaCl_2_.2H_2_O), disodium phosphate heptahydrate (Na_2_HPO_4_.7H_2_O), ethylenediaminetetraacetic acid disodium salt dihydrate (EDTA), heparin, magnesium chloride (MgCl_2_.6H_2_O), methylene blue, potassium chloride (KCl), potassium dihydrogen phosphate (KH_2_PO_4_), disodium basic phosphate (Na_2_HPO_4_), are all of analytical grade and were obtained from El-Nasr chemicals company (Abou-Zaabal, Cairo, Egypt).

### Biological resources

#### Ehrlich ascites carcinoma (EAC) cells

EAC-tumor is of a mammary origin. A spontaneous breast cancer served as the original tumor from which an ascites variant was obtained. An ascites rich in tumor cells was produced by IP inoculation. EAC cells were established in the Netherlands Cancer Institute. The Ehrlich-tumor cells were maintained in the laboratory of Faculty of Pharmacy, Mansoura University, in female Swiss albino mice by serial IP passage at 7–10 day intervals, as described earlier [7].

#### Animals and their treatment outlines

##### Mice

Female Swiss albino mice weighing 20–30 g were used to evaluate the anti-tumor activity of the natural compounds. Male mice were not used because of their poor tumor growth [8].

##### Rats

Male Sprague-Dawley rats weighing 110–160 g were used for evaluating the protection of the natural compounds against CP-induced nephrotoxicity.

The doses used for DHA in this study were in the range of those used in other studies applied for the same animals. These were determined after appropriate preliminary experiments. The time course of experiments in this study was in the range of those used in other studies applied for the same model of animals. This was determined after appropriate preliminary experiments.

#### Evaluation of the anti-tumor profiles of DHA in mice

This was performed by measuring the following parameters:

##### Monitoring of Tumor size

Female Swiss albino mice were inoculated with EAC cells and used for evaluating the anti-tumor effects of the natural agents under investigation. EAC cells were obtained, counted and diluted as described earlier [9]. After 7–8 days from EAC cell inoculation, ascites fluid was withdrawn from tumor-bearing mice by needle aspiration from the peritoneal cavity under aseptic conditions. Fluid was washed three times with normal saline by centrifugation at 1000 r.p.m at room temperature (RT). EAC cells were then tested for viability using trypan blue. The cells were examined microscopically using a hemocytometer. Following the viability test, cells were counted under the microscope. Then, they were suspended in normal saline so that each 0.1 mL contained 5 × 10^5 ^viable cells. Solid tumors were induced in mice by SC inoculation of 0.1 mL containing 5 × 10^5 ^viable tumor cells on the left flank anterior to the hind leg [10]. Tumor growth was determined by caliper measurement of the largest diameter and its perpendicular [11]. Tumor size was calculated as: Tumor size (mm^3^) = 0.5 × a × b^2^, where a: the largest diameter and; b is its perpendicular. When the primary tumor reached a size of 50–100 mm^3 ^(day 0), animals were divided into the following groups, each composed of 8–10 mice, as follows. All groups were injected with EAC cells except group (1):

▪ **Group (1): **(Normal-control group), received CMC, IP, daily, for 20 days from the 1^st ^day of the experiment (0.2 mL).

▪ **Group (2): **(EAC-control group), received CMC, IP, daily, for 20 days from the 1^st ^day of the experiment (0.2 mL).

▪ **Group (3): **Received CP (5 mg/kg, IP, single treatment on the 1^st ^day of the experiment), 1 h prior to the injection of CMC, IP, daily, for 20 days from the 1^st ^day of the experiment (0.2 mL) [12].

▪ **Group (4): **Received DHA (125 mg/kg, PO for 20 days from the 1^st ^day of the experiment).

▪ **Group (5): **Received DHA (250 mg/kg, [13]). It was given PO for 20 days from the 1^st ^day of the experiment.

▪ **Group (6): **Received CP (5 mg/kg, IP, single treatment on the 1^st ^day of the experiment) 1 h prior to the administration of DHA (125 mg/kg, PO for 20 days from the 1^st ^day of the experiment).

The anti-tumor activity was assessed by calculating the relative tumor volume (rTV) for each individual tumor by dividing the tumor volume on day 20 (V_20_) by the tumor volume on day 0 (V_0_) multiplied by 100% [14].

#### Lipid peroxides, measured as thiobarbituric acid reactive species (TBARs)

TBARs, mainly malondialdehyde (MDA) were measured according to the reported methods [15]. In this assay, thiobarbituric acid reacts with MDA in acidic medium at 95°C for 30 min. to form thiobarbituric acid reactive product. The absorbance of the resultant pink product can be measured at 534 nm. For this purpose, blood was collected and allowed to clot, then centrifuged at 2,000 r.p.m for 15 min. at 4°C. The top yellow serum layer was pipetted off without disturbing the white buffy layer. MDA was assayed in the same day. Linearity of the reaction was up to 100 nmol/mL.

#### Serum C-reactive protein (CRP) level

CRP was assayed according to the reported methods [16]. A kit obtained from Spinreact Co, Spain, is used. The CRP reagent is a suspension of polystyrene latex particles, coated with goat IgG anti-CRP. When CRP is present in the sample (mouse serum), the presence of agglutination indicates a level of CRP equal to or greater than 6 mg/L.

#### Leukocytic count

White blood cell (WBC) count was estimated using hemocytometer apparatus, according to the reported methods [17].

### The effect of DHA on CP-induced nephrotoxicity in rats

As reported in the literature, nephrotoxicity can be induced by injecting CP (7–10 mg/kg, IP, single dose [6,18]. In the present study, a dose of (10 mg/kg) was chosen after preliminary experiments. This evoked a significant increase in serum urea and creatinine levels after 7 days from the start of the experiment (3 days after CP injection). After 10 days, all the animals treated by CP alone died. Hence, the rats were divided into the following groups (8 rats each):

▪ **Group 1: **(Control group), received 0.4 mL of CMC, IP, for 7 days.

▪ **Group 2: **Received CP (10 mg/kg, IP, on the 4^th ^day of the experiment) 1 h prior to the injection of 0.4 mL of CMC, IP. The animals received 0.4 mL of CMC, IP, for 7 days from the 1^st ^day of the experiment.

▪ **Group 3: **Received CP (10 mg/kg, IP, on the 4^th ^day of the experiment) 1 h prior to the administration of DHA (125 mg/kg, PO, for 7 days from the 1^st ^day of the experiment).

▪ **Group 4: **Received CP (10 mg/kg, IP, on the 4^th ^day of the experiment) 1 h prior to the administration of DHA (250 mg/kg, [19]). DHA was given PO for 7 days from the 1^st ^day of the experiment.

▪ **Group 5: **Received CP (10 mg/kg, IP, on the 4^th ^day of the experiment) 1 h prior to the administration of DHA (250 mg/kg, PO, for 10 days from the 1^st ^day of the experiment).

▪ **Group 6: **Received DHA (250 mg/kg, PO, for 10 days from the 1^st ^day of the experiment).

After the end of the specified period of the experiment (7 or 10 days), the animals were anesthetized using ether, and then blood samples were collected, as mentioned previously, to separate serum samples for assaying urea and creatinine. Finally, the animals were sacrificed and kidneys were quickly removed and washed in ice cold isotonic saline, decapsulated, dissected, decorticated, weighed and minced. A 10% (w/v) homogenate was made in PBS (pH 7.4). The homogenate was centrifuged at 6000 r.p.m for 10 min. at 4°C and the supernatant was removed and stored on ice for assay of reduced glutathione (GSH) and lipid peroxides as MDA.

For the groups terminated after 7 days, survival studies were also performed using similar, but separate, groups treated for 10 days. This treatment period was judged by preliminary studies based on the survival of animals in the CP-group. The following parameters were evaluated:

#### Measuring kidney glomerular function

It was estimated through the following parameters:

##### Serum creatinine

Creatinine was measured in rat sera kinetically [20]. The assay depends on that creatinine in alkaline solution reacts with picrate to form a colored complex. The rate of complex formation is measured photometrically at 492 nm. A kit from Dp International Co, Egypt was used.

##### Serum urea

Urea was measured enzymatically in rat sera according to the reported procedures [21]. A kit from El-Nasr Co, Egypt was used.

##### Glomerular filtration rate

Glomerular filtration rate was estimated by measuring the renal plasma clearance of inulin (C_inulin_). Concentration of inulin was measured in urine and plasma samples after precipitation with 10% TCA; and clearance values were expressed in mL/min/100 g of initial body weight, according to the reported procedures [22].

##### Urine total protein (proteinuria)

The urine samples were collected over twenty four hours from rats placed in metabolic cages.

Urinary protein concentration was measured using the Lowry assay [23].

#### Measuring systemic inflammation, oxidative stress and the anti-oxidant potential in kidney homogenates

They were evaluated by measuring the following parameters:

##### Tumor necrosis factor-alpha (TNF-α)

TNF-α was quantitated by ELISA, using an ELISA assay (Quantikine Rat TNF-α kit; R&D Systems Inc., Minneapolis, Minnesota, USA). Kidney tissue was homogenized in PBS containing 0.05% Tween-20. Aliquots containing 300 μg of total protein were used for the TNF-α assay according to the protocol of the manufacturer.

##### Lipid peroxides measured as TBARs (MDA)

MDA was measured by the same method used in assaying serum MDA level in mice except that the sample was kidney homogenates. Homogenates were prepared right after the animals were killed and kept on ice. MDA was assayed in the same day.

##### Reduced glutathione (GSH)

It was measured in rat kidney homogenates according to the reported procedures [24]. The method is based on the reduction of 5,5'-dithiobis(2-nitrobenzoic acid) (DTNB) with GSH to produce a yellow compound. The intensity of color is directly proportional to GSH concentration and is measured at 405 nm. A kit obtained from Biodiagnostic Co, Egypt, was used to measure GSH. Linearity of the reaction is up to 120 mg/dL (4 mmol/L).

### Statistical analysis

Statistical significance between two groups was evaluated by Student's t-test for unpaired data. Comparison among multiple groups was conducted using one-way analysis of variance (ANOVA) followed by Tukey's post hoc test to determine significance among the means of the data groups. Linear regression equation was used for curve fitting and determination of the correlation coefficient "r". *X*^*2 *^test was applied to compare the survival number between different groups. Statistical significance was predefined at P < 0.05.

## Results

**Figure 1:**
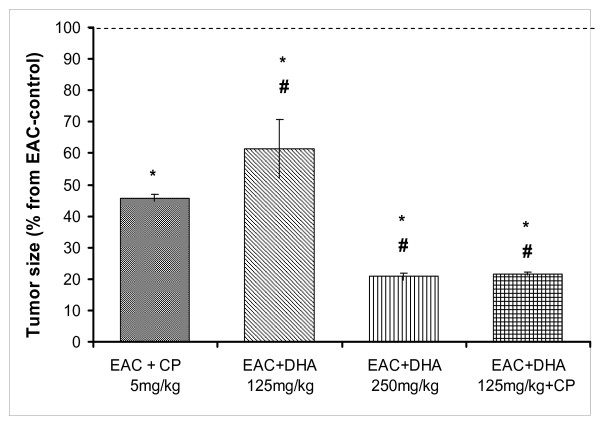
**Effect of DHA (125, 250 mg/kg/day) and/or CP (5 mg/kg/single dose) on the rate of EAC-solid tumor growth in mice**. Solid tumors were induced by SC inoculation of viable tumor cells on the left flank anterior to the hind leg till a tumor size of 50–100 mm^3 ^was obtained. Animals were then injected with the above drugs for 20 days. Tumor size was calculated as a percentage of relative tumor size (day 20/day 0) for each individual tumor. Data of different groups were normalized to that of the EAC-control group. Symbols indicate significance against; # CP-treated group; ° normal control group; and * EAC-control group.

**Figure 2:**
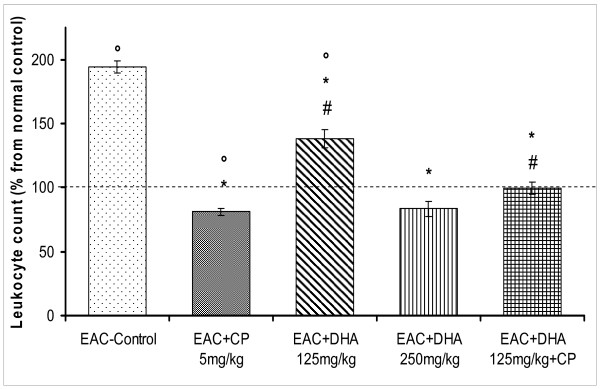
**(A) Effect of DHA (125, 250 mg/kg/day) and/or CP (5 mg/kg/single dose) on the EAC-induced leukocytosis in mice**. Solid tumors were induced and animals were treated as under Figure-1. After 20 days, blood was collected and leukocyte count was determined by means of a hemocytometer. Average counts of different groups were normalized to that of the normal control group. Symbols indicate significance against; # CP-treated group; ° normal control group; and * EAC-control group. (B): Correlation between leukocytic count and tumor size in EAC-solid tumor bearing mice that had been treated with DHA (125, 250 mg/kg/day) and/or CP (5 mg/kg, single dose). r denotes the correlation coefficient obtained for the linear regression line.

**Figure 3:**
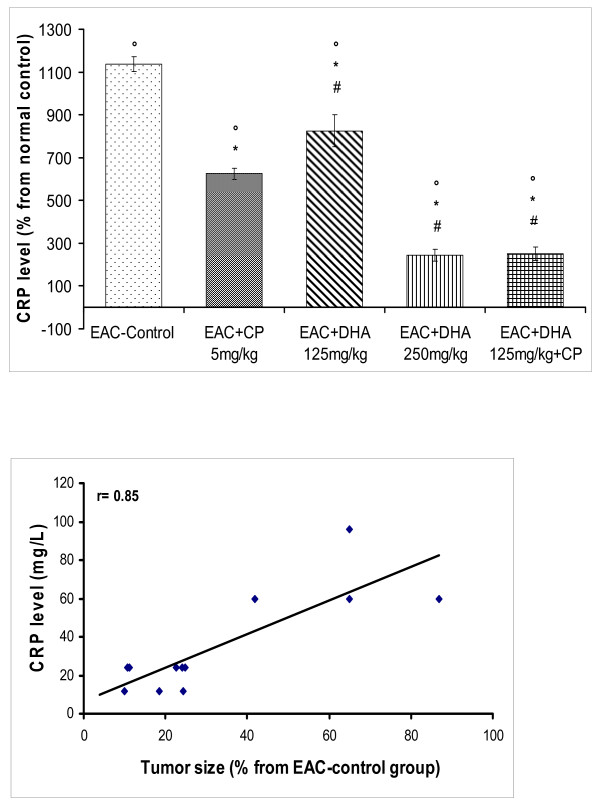
**(A) Effect of DHA (125, 250 mg/kg/day) and/or CP (5 mg/kg, single dose) on serum C-reactive protein (CRP) level in EAC-solid tumor bearing mice**. Solid tumors were induced and animals were treated as under Figure-1. After 20 days, blood was collected and sera separated for determining the level of CRP. Means of data for different groups were normalized to that of the normal control group. Symbols indicate significance against; # CP-treated group; ° normal control group; and * EAC-control group. (B): Correlation between CRP and tumor size in EAC-solid tumor bearing mice that had been treated with DHA (125, 250 mg/kg/day) and/or CP (5 mg/kg, single dose). r denotes the correlation coefficient obtained for the linear regression line.

**Figure 4:**
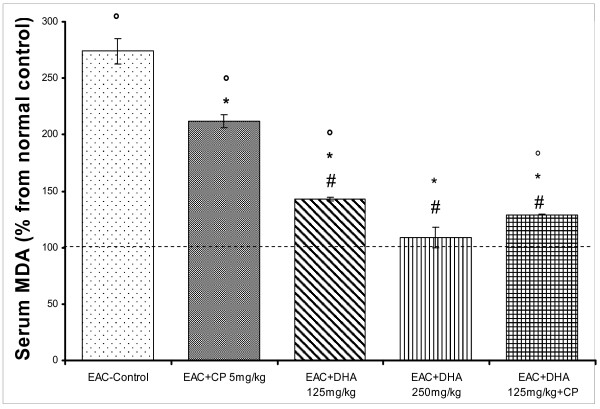
**Effect of DHA (125, 250 mg/kg/day) and/or CP (5 mg/kg, single dose) on serum lipid peroxide level (measured as MDA) in EAC-solid tumor bearing mice**. Solid tumors were induced and animals were treated as under Figure-1. After 20 days, blood was collected and sera separated for determining the level of MDA. Average data of different groups were normalized to that of the normal control group. Symbols indicate significance against; # CP-treated group; ° normal control group; and * EAC-control group.

Induction of solid tumor in mice was accompanied by marked cellular, molecular, and biochemical changes that included leukocytosis, elevation of serum c-reactive protein (CRP), and enhanced lipid peroxidation. The latter free radical was measured as malondialdehyde; MDA. These parameters were utilized as sensors for the extent of tumor development and/or DHA-induced chemoprevention (Figures 1, 2, 3, 4). Thus, relative to normal control mice, inoculation of EAC-cells led to the development of a solid tumor after 2 weeks. Treatment with the cytotoxic drug, cisplatin (CP, 5 mg/kg) led to a 55% reduction in tumor size (p < 0.05). Likewise, treatment with DHA (125 mg/kg) evoked a 38% inhibition in tumor growth (p < 0.05), a response that was; however, significantly lower than that of CP (p < 0.05). By contrast, a higher dose of DHA (250 mg/kg) strikingly suppressed tumor growth, by 79%, (p < 0.05); a cytotoxic response that appreciably surpassed that produced by CP (p < 0.05). Combining the lower dose of DHA (125 mg/kg) with CP significantly enhanced the chemopreventive effects of both drugs than either of their individual responses (81% inhibition of growth, P < 0.05). Figure 2A indicates that EAC evoked a 2-fold rise in leukocytic count, a primary antecedent inflammatory response in the process of carcinogenesis. CP evoked reduction from both the normal control (leukopenia) and the EAC-control group. The lower DHA dose (125 mg/kg) reduced leukocytosis from that of the solid tumor level, yet it still remained higher than either of the CP or the control levels (p < 0.05). Increasing DHA to 250 mg/kg also reduced the EAC-induced leukocytosis virtually to the baseline level (P < 0.05). When the low dose DHA was combined with CP, both drugs reduced the leukocytic count to the baseline level, thus avoiding leukopenia seen with CP. Correlation studies that utilized data obtained for tumor-size and leukocytosis (Figure 2B) suggest a high degree of a significant positive association between the two phenomena (r = 0.89).

The diagnostic/prognostic role of C-reactive protein (CRP) has been sought in some types of cancer; though reports are still divisive [25]. Here, EAC brought about almost 11-fold rise in that protein level. This response was attenuated by CP to 6.0-fold of control level (P < 0.05). DHA (125 mg/kg) also reduced CRP level (P < 0.05) than that of the EAC-group, down to 8-fold above the baseline. This remained however significantly less potent than the CP response (P < 0.05). Notably, a more substantive reduction, down to 2.5-fold, was obtained by the higher DHA dose (250 mg/kg). The latter response was reproducibly achieved by combining CP with the low dose DHA (125 mg/kg). To validate the significance of this protein in evaluating tumor progression or responsiveness to cytotoxic agents, correlation curve was constructed between tumor size and CRP level for the various mice-treated groups. As can be seen in figure 3B, a high positive association linear regression curve was produced (r = 0.85). On the other hand, the level of lipid peroxides (LPO), in terms of MDA, was estimated as a measure of oxidative stress in mouse sera (Figure 4). MDA significantly rose along with the induction of EAC-solid tumor (2.75 fold). Though this level was reduced significantly by CP (down to 2.0-fold, P < 0.05), it was still markedly higher than the baseline (P < 0.05). DHA (125 mg/kg) showed a better response than CP (p < 0.05), thus lowering MDA to a 0.5 fold above baseline (P < 0.05). Animals that received higher DHA dose (250 mg/kg) had LPO levels similar to those of the control animals. Correlation studies between MDA and tumor size, showed insignificant positive association between the two parameters (r = 0.52; data not shown).

**Figure 5:**
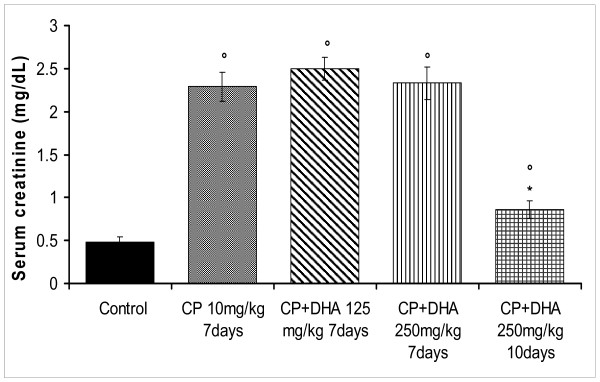
**Effect of DHA (125, 250 mg/kg/day) on CP (10 mg/kg, single dose)-induced renal dysfunction in rats**. DHA was given throughout day1-day7 (or day1-day10) of the experiment; depending on animal survival. CP was given on day4. Blood was collected and sera separated for measurements of creatinine (5A) and urea (5B) levels, after 7 or 10 days; depending on animal survival. Data of different groups are expressed as mean +/- SEM of individual animal levels. Symbols indicate significance against; respective *CP-treated group; and °control group.

**Figure 6:**
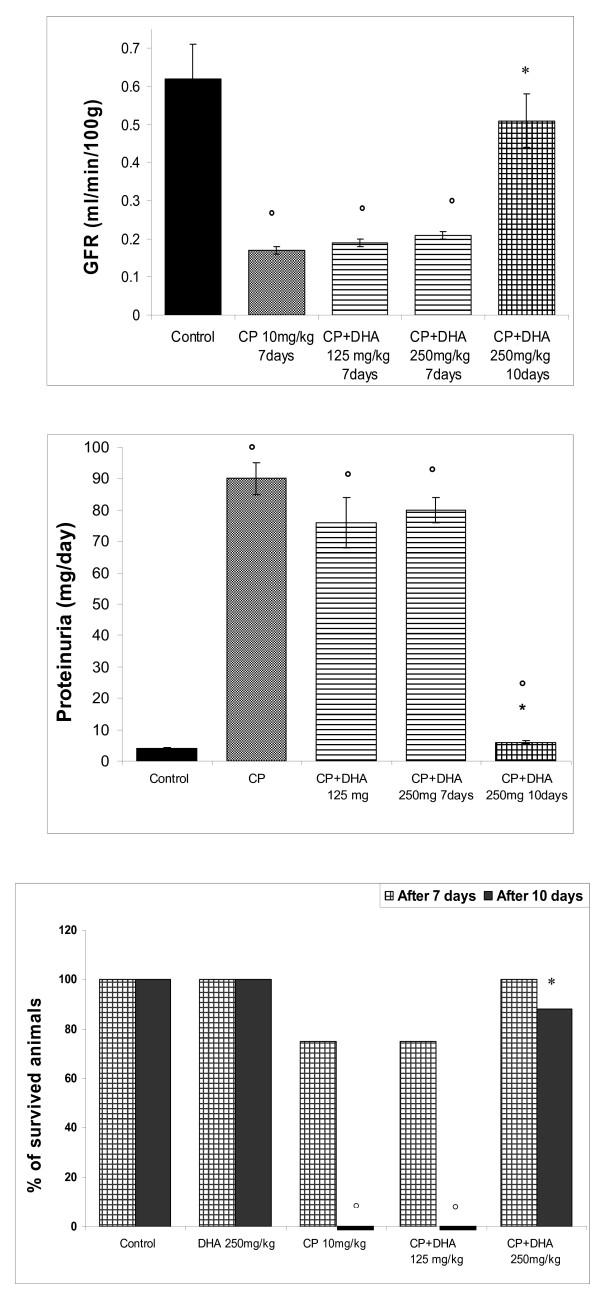
**Effect of DHA (125, 250 mg/kg/day) on CP (10 mg/kg, single dose)-induced impairment of GFR (6A), and proteinuria (6B) in rats**. DHA was given throughout day1-day7 (or day1-day10) of the experiment; depending on animal survival. CP was given on day4. Data of different groups are expressed as mean +/- SEM of individual animal levels. 6C shows animal survival after 7 and 10 days following different drug-treatments. (6C): survived animal % after 7 and 10 days of different drug-treatments. Symbols indicate significance against; respective *CP-treated group; and respective °control group.

A seriously known adverse reaction to CP in clinical settings is its nephrotoxicity, an also well-established drug-induced nephrotoxicity animal model. Thus, a single CP dose (10 mg/kg, injected IP, injected on the 4^th ^day) induced lethal nephrotoxicity in rats after a total of 7 days. This was indicated by disruption of renal function, as reflected by substantially elevated serum levels of urea (5 fold) and creatinine (2.5 fold) after 7 days (P < 0.05) (Figures 5A, 5B). This was accompanied by a global animal fatality after 10 days. At day 7, animals that had received DHA (125 mg/kg) or (250 mg/kg) did not show improved glomerular filtration rates (Figure 6A) and had persistently mounting proteinuria (Figure 6B). However, as evident from figure 6-C, resuming treatment for 10 days rescued 88% of the rats that only received the higher DHA dose (250 mg/kg). This was also consonant with an appreciable reduction in levels of both urea and creatinine, as compared to the CP-treated rats (Figures 5A, 5B). Control animals that had received only DHA (without CP) showed no change in their renal function tests nor were their survival threatened as well.

**Figure 7:**
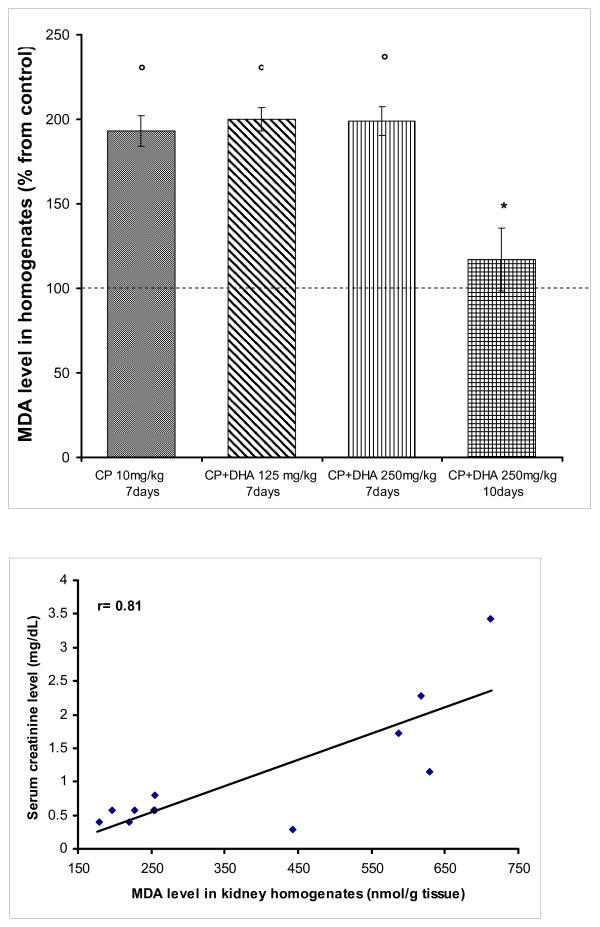
**(A) Effect of DHA (125, 250 mg/kg/day) and CP (10 mg/kg, single dose) on MDA level in rat kidney homogenates**. DHA was given throughout day1-day7 (or day10) of the experiment; depending on animal survival. CP was given on day4. Animals were sacrificed, kidneys removed and homogenates prepared. Data represent means +/- SEM of individual MDA levels in different groups, expressed as a percentage of the control group. Symbols indicate significance against; *CP-treated group; and °control group. (B): Correlation between MDA and creatinine levels in rat kidney homogenates treated with CP (10 mg/kg, single dose) and/or DHA (125, 250 mg/kg/day). r denotes the correlation coefficient obtained for the linear regression line.

**Figure 8:**
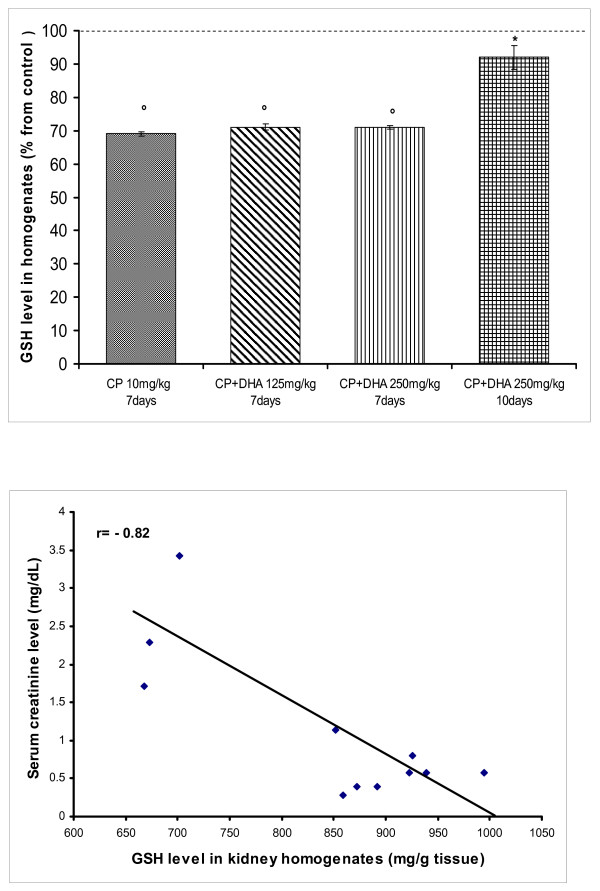
**(A) Effect of DHA (125, 250 mg/kg/day) and CP (10 mg/kg, single dose) on reduced glutathione (GSH) level in rat kidney homogenates**. DHA was given throughout day1-day7 (or day10) of the experiment; depending on animal survival. CP was given on day4. Animals were sacrificed, kidneys removed and homogenates prepared. Data represent means +/- SEM of individual GSH levels in different groups, expressed as a percentage of the control group. Symbols indicate significance against; *CP-treated group; and °control group. (B): Correlation between GSH and creatinine levels in rat kidney homogenates treated with CP (10 mg/kg, single dose) and/or DHA (125, 250 mg/kg/day). r denotes the correlation coefficient obtained for the linear regression line.

Oxidative stress is a hallmark of CP-induced nephrotoxicity [26]. Also, DHA is a polyunsaturated fatty acid with reportedly inconsistent effects on LPO [5]. Hence, we focused on evaluating the effects of DHA on MDA, as well as on reduced glutathione (GSH; a crucial endogenous anti-oxidative/membrane preserving thiol) in kidney homogenates from variously treated rats. Figure 7A points out that after 7 days of treatment, CP almost doubled the level of MDA in rat kidney homogenates. This responsiveness remained unchanged when CP was combined with DHA (125 or 250 mg/kg) for this treatment period. Resuming DHA treatment for 10 days, however, resulted in a remarkable reduction in MDA level to near control levels (p < 0.05 against CP). To gain insights into possible causal relationships of lipid peroxidation in disruption of GFR, correlations were made between MDA and creatinine levels. Figure 7B demonstrates that a considerable positive association exists between the MDA level and creatinine, in response to various drug treatments (r = 0.81). This indicates that reduction of renal MDA level contributes to renal function protection. On the other hand, treatment with CP significantly depleted (GSH) level in kidney homogenates. Concurrent treatment with DHA (250 mg/kg) restored GSH to near normal concentrations in kidney tissues (p < 0.05; against CP) (Figure 8A). Further, a high degree of negative association between GSH and creatinine levels was observed (Figure 8B, r = -0.82), thus confirming an active role for GSH in the mechanisms governing both renal tissue injury by CP (lower levels), and protection therefrom by DHA (higher levels).

**Figure 9:**
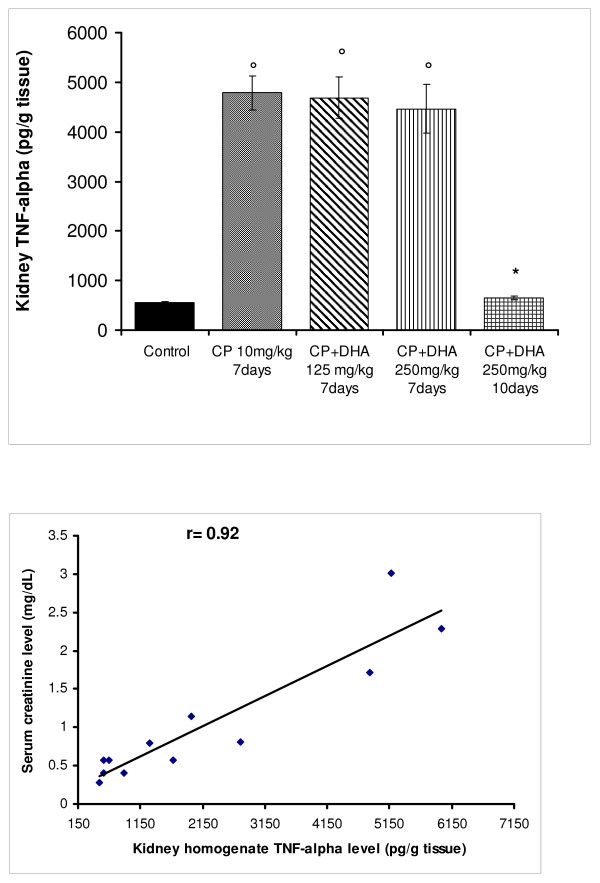
**(A) Effect of DHA (125, 250 mg/kg/day) and CP (10 mg/kg, single dose) on TNF-α level in rat kidney homogenates**. DHA was given throughout day1-day7 (or day10) of the experiment; depending on animal survival. CP was given on day4. Animals were sacrificed, kidneys removed and homogenates prepared. Data represent means +/- SEM of individual TNF-α levels in different groups. Symbols indicate significance against; *CP-treated group; and °control group. (B): Correlation between TNF-α and creatinine levels in rat kidney homogenates treated with CP (10 mg/kg, single dose) and/or DHA (125, 250 mg/kg/day). r denotes the correlation coefficient obtained for the linear regression line.

Renal inflammation is a primary antecedent in impaired renal diseases; as currently exemplified by the CP-evoked nephrotoxicity [2]. Accordingly, we assessed levels of TNF-α, a marker of systemic inflammation, in renal tissues treated with and without CP, DHA and their combinations. Figure 9A shows that CP evoked ~10-fold rise in renal tissue TNF-α levels after 7 days. Co-treatment for this same period with DHA (either 125 or 250 mg/kg) failed to effectively reduce TNF-α levels from those attained with CP-group. Conversely; however, extending the co-treatment period with 250 mg/kg of DHA for 10 days blunted the rise in TNF-α levels (p < 0.05). Figure 9B further demonstrates that the rise in TNF-α level (inflammation) highly correlated with that of serum creatinine (renal dysfunction) (r = 0.92). These findings suggest that TNF-α is both a key trigger of nephrotoxicity in response to CP treatment, and a crucial target in alleviation of such anomalies by DHA.

This study was the first to seek the anti-tumor capacity of DHA on EAC-solid-tumor model in mice. Mice were treated with two doses of DHA (125, 250 mg/kg) for 20 days, either alone or in combination with CP (5 mg/kg, single treatment). DHA alone showed anti-tumor activity, indicated by a significant reduction in tumor size, as compared to the EAC-control group. The higher dose of DHA (250 mg/kg) evoked a greater anti-tumor activity, indicating that the anti-tumor activity of DHA is dose-dependent. The combination of DHA with CP boosted the anti-tumor capacity of CP, as indicated by a more significant reduction in tumor size, relative to the CP-group. This was in agreement with earlier findings which showed that DHA enhanced the activity of some anti-cancer drugs, such as anthracyclines [4]. It also appears that DHA improves the overall biological profiles of CP; as seen with leukocytosis in this study where a 2 fold-rise was obtained. Leukocytic count often increases in cancer patients, and has been known as a paraneoplastic syndrome that occurs occasionally in patients with oral malignancies [27]. Albeit CP reduced leukocytosis in our EAC-model, it further led to a marked leukopenia; which goes well with the known bone marrow suppressive potential known for CP. This leukopenia was abolished when DHA was jointly given with CP. This concerted action therefore would help maintain the integrity of the immune system.

The obviously reported positive association between cancer and inflammation suggests a potential role for CRP and leukocytosis as possible indicators of cancer development. However, until very recently, a paucity of information was available as to whether CRP can serve as a prognostic or diagnostic factor in cancer. This information was also often inconsistent and appear to be cancer-type specific. For instance, some studies provided a modest evidence for a positive diagnostic role of CRP in cancer [28]. Conversely, some researchers found that elevated plasma CRP concentration appears to be a strong predictor of poor survival and lower probability of prostate-specific antigen response to treatment in patients with androgen-independent prostate cancer [29]. The CRP role as a predictor of survival has been shown in multiple myeloma, melanoma, lymphoma, and various ovarian-, renal-, pancreatic-, and gastrointestinal-tumors [25]. Others suggested that the pre-operative serum elevation of both IL-6 and CRP are good prognostic predictors in colorectal cancer patients [30]. In addition, a few studies reported that some tumor-based events, such as expression of IL-6 receptor and COX-2 enzyme, or infiltration of T-lymphocytes, are inferior to CRP in determining survival in patients with localized renal cell carcinoma [31].

Accordingly, this work developed an interest to study the correlation between CRP level and the progression of EAC-solid-tumor. Here, it was the first time ever to study the correlations between tumor size, leukocytic count and serum CRP level in the EAC-solid-tumor model. We found that leukocytic count and CRP level were significantly increased in response to a mount in tumor size. Further, treatment with CP and DHA showed a significant reduction in the tumor size that was paralleled with a fall in both CRP level and leukocytosis. The correlation curves we constructed indicated a high degree of positive association between tumor size and both of CRP level and leukocytic count (r = 0.85 and 0.89; respectively), thus supporting our hypothesis that CRP may well serve as a prognostic and/or diagnostic factor in this tumor model. Thus, both the extent of tumor progression, as well as the prediction of responsiveness to chemotherapy may well be inferred through CRP measurements.

Overall, the *in vivo *epidemiological and experimental studies have not yet clearly conferred a strong evidence for clinically beneficial effects of ω-3 polyunsaturated fatty acids (PUFAs) in breast cancer. However, some evidence was revealed by *in vitro *studies or by limited clinical work that ω-3 PUFAs may reduce the risk of breast cancer development [32]. A more recent study indicated that ω-3 PUFAs decrease cell proliferation and induce apoptotic cell death in human breast cancer cells, possibly by decreasing signal transduction through the Akt/NF-κB cell survival pathway [33]. In addition, dietary intake of ω-3 PUFAs was shown to reduce growth and metastasis of mammary tumors [34]. Because EAC is a breast-cancer-related tumor, our present results favor a promising profile for DHA against breast cancer, at least when combined with traditional chemotherapeutic agents like CP.

On the other hand, the selectivity of DHA and other PUFAs on tumor cells has been a worthwhile goal of research. In this context, *in vitro *cell culture studies have led to a consensus that some PUFAs exert a selective cytotoxic or anti-proliferative effect on tumor cells rather than on normal cells [35]. The cellular protective effects of DHA were also investigated by some studies. Thus, DHA was found to protect neuronal cells from apoptosis by inhibiting the activity of caspase enzyme [36].

On the contrary, several studies have been divisive over the ultimate effects of DHA/PUFAs on lipid peroxidation and its sequaleae. These FAs, themselves, can undergo peroxidation with free oxygen, to produce lipid hydroperoxides, which in turn propagate further peroxidation into peroxy and alkoxy radicals. The latter compounds may break down to cyclic endoperoxides and MDA-like products. LPO is capable of affecting cell proliferation due to the formation of inter- and intra-molecular linkages between amino acid sulfhydryl groups of RNA, DNA and proteins, leading to damage to these molecules. The LPO products stimulate DNA repair processes and activate polymerase leading to the depletion of NAD and ATP. When peroxidation damage exceeds the repair process, it will finally lead to cell death [37]. Many cancer cells were killed by the PUFA-generated ROS, although the killing mechanisms varies based on the cell type involved. Higher concentrations of PUFAs increase the sensitivity to ROS in tumor cells because of the cytotoxicity of LPO and its metabolites [38]. Hence, because our studies were performed *in vivo*, and MDA was measured in serum, a direct link would be difficult to be drawn. Thus, some previous studies have indicated an increase in ROS with DHA administration [39]. However, in the present study, we found a significant decrease in serum MDA levels in the animals treated with DHA, either alone or in combination with CP, as compared to the EAC-control group. This was in agreement with earlier studies [40]. This reduction in MDA level in groups treated with DHA may be explained on the basis that the free radicals produced at site of action induce a marked, systematic anti-oxidant response as a feedback to oxidative stress. This increase in anti-oxidants would augment anti-oxidative potency in tissues and thus eventually decrease LPO, resulting in the suppression of end products of LPO, such as MDA. These prepositions are in line with others that showed protective effect for DHA on LDL oxidizability [41]. In addition, DHA was found to suppress the expression of iNOS gene, and the production of NO [42]. Our finding that DHA enhanced GSH levels in renal tissues may support this view as well. It is also important to notice that we did not find a significant positive association between serum MDA levels and tumor size in this study. This suggested that serum levels of MDA, *in vivo*, may well vary from those obtained in tumor cells through *in vitro *studies. It is not also unreasonable to assume that interaction of PUFAs or their metabolites with additional signaling pathways could contribute to their *in vivo *chemopreventive effects [43].

In the present study, a single CP dose (10 mg/kg, injected IP on the 4^th ^day) induced lethal nephrotoxicity in rats after a total of 7 days. Because of the wide use of CP in clinical settings, several pathological and toxicological studies have investigated the underlying mechanisms of such CP-induced nephrotoxicity. It was generally agreed that the platinum content of CP is the main trigger of these anomalies. Kidneys tend to accumulate and retain platinum complexes to a greater extent than other organs, and it is also the main excretory outlet for CP when given by the IV or IP routes [44]. Ultimately, these events cause renal tubular damage and dysfunction, proteinuria, and wasting of sodium, potassium, and magnesium. Notably, approximately 25–35% of patients develop evidence of nephrotoxicity following a single dose of cisplatin [45]. On the contrary, the exact cellular and molecular underpinnings of such CP-evoked nephrotoxicity remain largely to be unfolded. The reason of this ambiguity is that CP interacts with several intracellular pathways. Examples encompass gene modulations, activation of mitogen-activated protein kinases, induction of apoptosis, stimulation of inflammation and fibrogenesis, depletion of L-carnitine, and induction of cytotoxicity by promoting the accumulation of ROS. Thus, out of this plethora of reactions, the adoption of an exact scenario appears to be a difficult task [45]. Nevertheless, evidence from both *in vitro *and *in vivo *studies was presented to testify for a prime contribution of reactive oxygen metabolites in the pathogenesis of this nephrotoxicity. In this vein, CP was shown to generate ROS, such as superoxide anion and hydroxyl radicals, and stimulate renal LPO accumulation [26]. The role of LPO and its position in the chain of events that leads to CP nephrotoxicity remains, however, controversial.

One of the most important intracellular anti-oxidant defense systems is the glutathione redox cycle. Reduced glutathione (GSH) is an essential component in maintaining cell integrity because of its reducing properties as well as its contribution to some essential cellular metabolic pathways [46]. Many previous studies showed that CP consistently depleted GSH, while elevated MDA levels, as compared to baseline levels [47-49]. However, other studies found that CP did not cause any significant change in kidney levels of MDA, yet still caused a significant elevation in endogenous GSH content of the kidney [50]. Therefore, this work represented an intriguing challenge for DHA to alleviate this CP-evoked nephrotoxicity. Our rational was based on reported cellular protective ability for DHA in some models. For instance, DHA supplement improved spatial cognition in rats, following transient forebrain ischemia [51]. Also, in the renal system, PUFA were found to protect against the renal tubular injury caused by shiga toxin. The latter toxin can modulate certain intracellular pathways, thereby inducing cell death [52]. Likewise, DHA inhibited mesangial cell activation and proliferation, and reduced proteinuria, two primary antecedents in the development of glomerular damage [53]. In addition, DHA significantly alleviated the doxorubicin-induced apoptosis of renal cortex cells [54]. The present study was, however, the first to describe protective effects for DHA against CP-induced nephrotoxicity in rats. DHA (250 mg/kg; but not 125 mg/kg), given prior to and concurrently with CP, attenuated CP-induced nephrotoxicity, improved renal glomerular function, and obliterated almost 90% of animal fatalities. Also currently, remarkable anti-oxidant potential was produced by DHA in the kidney homogenates. Thus, GSH level was significantly increased; and MDA level was decreased from CP-treated animals. Further, the results of correlations we made between the levels of MDA or GSH in kidney homogenates, and kidney glomerular function in terms of serum creatinine strongly implicate oxidant stress in mediating this type of nephrotoxicity (r = 0.81, -0.82).

## Conclusion

The present study assessed the chemopreventive potential of docosahexaenoic acid (DHA) and its impact on that of CP in the EAC-implanted solid tumor model in mice. Besides, it examined the mediatory role of oxidative stress, represented by lipid peroxides (LPO) and the possible diagnostic/prognostic role of CRP in the process of tumorigenesis. Lastly, because of the pronounced CP-induced nephrotoxicity in mammals, we also probed whether (and how) the concurrent administration of DHA would alleviate such serious adverse reaction to CP. The chemoprevention elicited by DHA was dose-dependent, and appear to be mediated by reduction of leukocytosis, oxidative stress, and replenishing of endogenous antioxidant machinery. Most strikingly, a strong anti-inflammatory effect was produced that was highly reflected by reduction in CRP level. We provided a novel, strong evidence that CRP serves as a sensitive diagnostic/prognostic marker both for tumor progression and responsiveness to chemotherapy in solid tumors.

In ischemic acute renal injury, inflammatory pathways appear to play an important pathogenic role, and a high level of cross-talk seems to exist between oxidant stress and inflammation in these settings [2]. For instance, oxidant stress, as in cisplatin-induced injury, activates the NFκB transcription factor, which in turn promotes the production of proinflammatory cytokines, including TNF-α [55]. We currently confirmed this signaling system and further challenged the capacity of DHA to downregulate this pathway. DHA 250 mg/kg managed to blunt the increment in TNF-α evoked by CP to near control values and rescued 90% of the animals from the evidently lethal CP nephrotoxicity. These findings correlated very well with data for GFR and creatinine levels (r = 0.92); thus attesting to a new mechanism of renal protection by DHA.

It is worthwhile verifying the clinical relevance of the current findings on the basis of comparing the DHA doses used in rat and mice to those used in human clinical trials. In the latter studies, DHA has been used in a range of 26–56 mg/kg/day [56]. These doses, based on dosage conversion schemes, are equivalent to (165–347 mg/kg/day) in rats and (320–690 mg/kg/day) in mice [57]; thus quite comparable to the range used in this study (125–250 mg/kg/day). Therefore, based on the animal/human dose relevance, and clinical studies for the chemo-enhancing profiles of DHA in humans, the current findings suggest a clinical chemopreventive/renal protective significance.

In essence, the renal protective and animal rescuing effects obtained for DHA, as well as its capacity to ameliorate leukocytosis, oxidant stress and inflammation, further substantiate its produced chemoprevention/chemo-enhancing profiles. This could, therefore, suggest a new fruitful drug regimen in the management of solid tumors based on combining CP with DHA. Similar studies for DHA with other antineoplastic drugs are also warranted.

## Competing interests

The authors declare that they have no competing interests.

## Authors' contributions

MEE, an MSc student, performed the lab work; also contributed to drafting of the manuscript/statistical analyses. MMA contributed to the oxidative stress and renal studies. HAS contributed to the pharmacological studies and their interpretation. MMD provided excellent technical support and contributed to analysis of many experiments. AME designed the plans, experiments, provided lab guidance and supervised the entire team towards appropriate interpretations of the data and their analyses. He further wrote up the manuscript, and managed its editorial correspondence, revisions, and finishing. All authors read and approved the MS.
